# Conjunctive Visual Processing Appears Abnormal in Autism

**DOI:** 10.3389/fpsyg.2018.02668

**Published:** 2019-01-18

**Authors:** Ryan A. Stevenson, Aviva Philipp-Muller, Naomi Hazlett, Ze Y. Wang, Jessica Luk, Jong Lee, Karen R. Black, Lok-Kin Yeung, Fakhri Shafai, Magali Segers, Susanne Feber, Morgan D. Barense

**Affiliations:** ^1^Department of Psychology, Western University, London, ON, Canada; ^2^Brain and Mind Institute, Western University, London, ON, Canada; ^3^Department of Psychiatry, Schulich School of Medicine and Dentistry, Western University, London, ON, Canada; ^4^Neuroscience Program, Schulich School of Medicine and Dentistry, Western University, London, ON, Canada; ^5^Centre for Vision Research, York University, Toronto, ON, Canada; ^6^Department of Psychology, The Ohio State University, Columbus, OH, United States; ^7^College of Occupational Therapists of Ontario, Toronto, ON, Canada; ^8^Department of Psychology, University of Toronto, Toronto, ON, Canada; ^9^Department of Psychology, York University, Toronto, ON, Canada; ^10^Rotman Research Institute at Baycrest, Toronto, ON, Canada

**Keywords:** face processing, autism spectrum disorder, visual processing, sensory, vision, holistic, object recognition

## Abstract

Face processing in autism spectrum disorder (ASD) is thought to be atypical, but it is unclear whether differences in visual conjunctive processing are specific to faces. To address this, we adapted a previously established eye-tracking paradigm which modulates the need for conjunctive processing by varying the degree of feature ambiguity in faces and objects. Typically-developed (TD) participants showed a canonical pattern of conjunctive processing: High-ambiguity objects were processed more conjunctively than low-ambiguity objects, and faces were processed in an equally conjunctive manner regardless of ambiguity level. In contrast, autistic individuals did not show differences in conjunctive processing based on stimulus category, providing evidence that atypical visual conjunctive processing in ASD is the result of a domain general lack of perceptual specialization.

## Introduction

The human face conveys a wealth of socially relevant information, notably information needed to distinguish one face from another. Although not a defining characteristic of the disorder, autistic individuals often present with deficits in the perception and recognition of face identity (for review, see Weigelt et al., 2012). There are at least two possible explanations for these difficulties. One is that face recognition difficulties are directly related to the *social* impairments observed in autism spectrum disorder (ASD). Under this view, one would predict selective impairments in processing *only* social stimuli such as faces and voices, with non-social stimuli left unaffected. There is some evidence to support this prediction. For example, face-voice pairs are processed atypically in autism, yet non-social stimuli are processed typically (Bebko et al., 2006; de Boer-Schellekens et al., 2013; Woynaroski et al., 2013; Stevenson et al., 2014a, 2015, 2017a,b; Wallace and Stevenson, 2014; Baum et al., 2015; Herrington et al., 2015; Noel et al., 2018).

Alternatively, difficulties in face recognition may stem from domain-general differences in *visual* processing that disproportionately affect face recognition. More specifically, faces are typically processed conjunctively. When perceiving a face, one does not process the component features of a face individually (e.g., the eyes, ears, nose, and mouth), but instead processes these individual features as a single, unified whole (Tanaka and Farah, 1993; Rossion, 2008; Van Belle et al., 2010; Behrmann et al., 2015). Difficulties in face recognition may result from domain-general impairments at the level of conjunctive processing, regardless of the socialness of the stimulus. Indeed, difficulties with conjunctive processing in autism have been demonstrated (Hobson et al., 1988; Joseph and Tanaka, 2003; Teunisse and de Gelder, 2003; Behrmann et al., 2006; Rose et al., 2007; Scherf et al., 2008; Faja et al., 2009), though not unequivocally (Tanaka and Sung, 2016). Additionally, this ability to bind individual pieces of sensory information to form unified percepts has been seen quite broadly both within and across sensory modalities (for review, see Baum et al., 2015).

**FIGURE 1:**
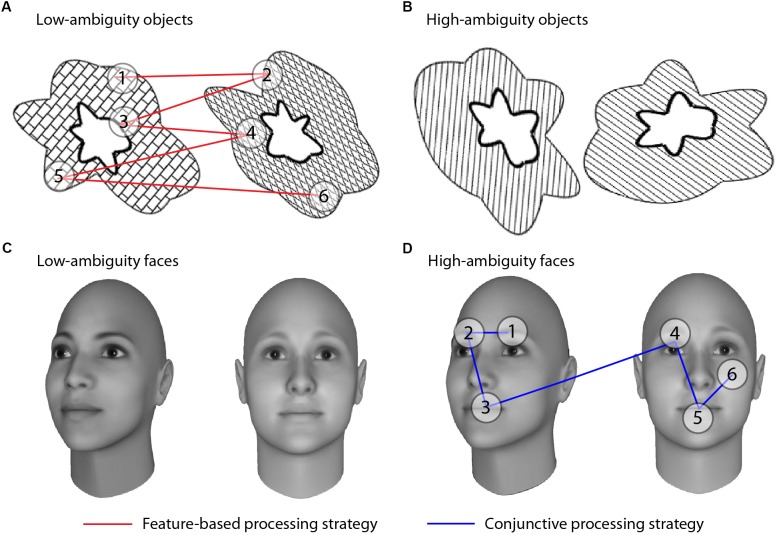
Visual discrimination tasks and processing strategies. Participants indicated whether stimuli were a match or a non-match. Stimuli consisted of abstract objects **(A,B)** and faces **(C,D)** manipulated to be low ambiguity **(A,C)** or high ambiguity **(B,D)**. All examples depict non-match trials; match trials comprised two identical, but rotated, stimuli. Conjunctive versus feature-based strategies were assessed by comparing the number of saccades an individual made within an object/face versus the number of saccades between objects/faces in non-match trials. If participants viewed the objects as a unified conjunction of features, we would expect more saccades *within* an individual object relative to saccades *across* the two objects (blue lines). If participants treated the objects as three separate individual features, we would expect to see more comparisons of features across the two objects than within an individual object (red lines). Each fixation is shown by a numbered circle indicating the order of the fixation; lines connecting the fixations indicate saccades.

To test these two alternative hypotheses of face processing differences in autism, we adapted an eye-tracking paradigm designed to parse conjunctive and feature-based visual processing that has previously been used with other clinical populations (Barense et al., 2012; Newsome et al., 2012). This paradigm exploits the fact that individuals process stimuli with visually overlapping features using a more conjunctive approach, relative to stimuli with less visual overlap. This property of visual discriminations has been termed “feature ambiguity;” a feature is said to be “ambiguous” when it is present on multiple objects and thus no longer uniquely predicts an object’s identity (Bussey et al., 2002, 2005; Bartko et al., 2007). When two stimuli are more ambiguous, or share more individual features, it becomes necessary to use conjunctions of features to discriminate between them (Bussey et al., 2002, 2005; Barense et al., 2005, 2007; Bartko et al., 2007; Erez et al., 2013). We thus presented participants with high- and low-ambiguity face and non-face stimuli while tracking their eye movements, allowing us to determine whether typically-developed (TD) and autistic individuals used conjunctive or feature-based processing strategies, and whether differences were specific to faces or also observed for non-face stimuli (Figure 1).

**FIGURE 2:**
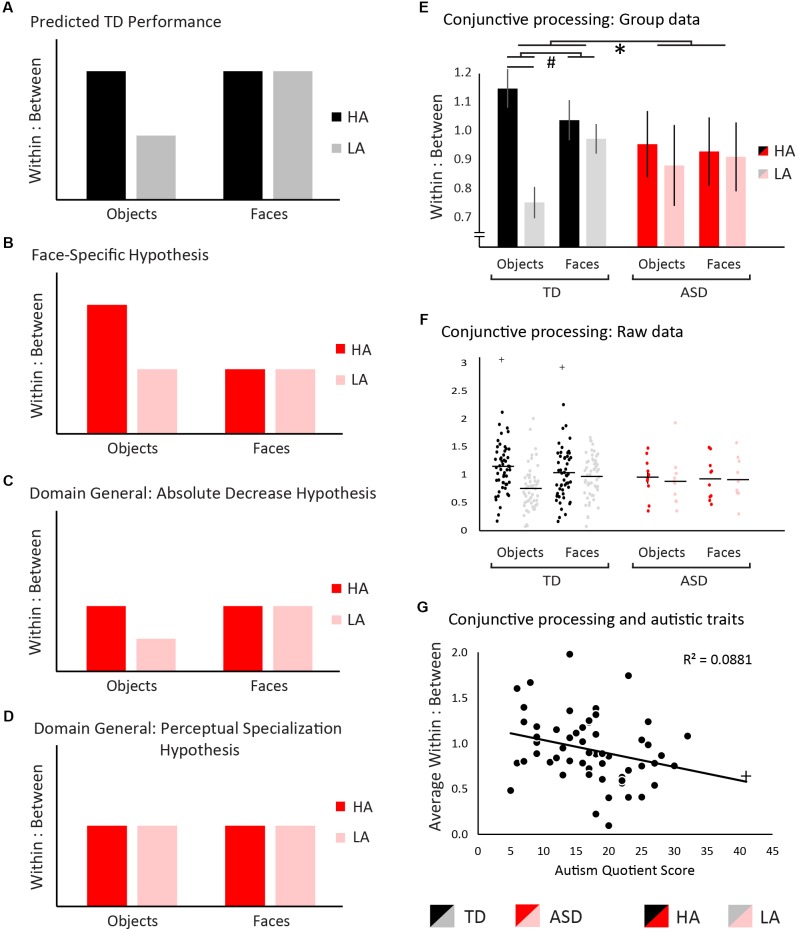
Hypotheses and results. Measurements of within:between saccade ratios were used to measure visual conjunctive processing, where higher ratios are indicative of a more conjunctive processing strategy, and lower ratios of a more feature-based strategy. **(A)** We predicted that typically-developed individuals would show greater conjunctive processing for high- relative to low-ambiguity objects, as well as high levels of conjunctive processing for both high- and low-ambiguity faces across ambiguity. **(B)** If the autism spectrum disorder (ASD) group shows a face-specific difficulty in conjunctive processing, conjunctive processing would be typical for objects, but decreased for faces. **(C)** If the ASD group exhibits a domain-general difficulty in conjunctive processing such that visual conjunctive processing is decreased overall, the within:between ratios would be reduced relative to TD, but their overall pattern would remain consistent across groups. **(D)** If the ASD group exhibits a domain-general difficulty in conjunctive processing through a lack of perceptual specialization, the pattern of within:between saccades would change such that the level conjunctive processing would not vary between any conditions and would be reduced overall relative to TD. For all panels, TD individuals are shown in black/gray and ASD individuals in red/pink. Panel **(E)** shows data for within versus between item saccades in the TD group and ASD group. Significant statistical effects are denoted with ^∗^ indicating a 3-way interaction, # indicating 2-way interactions, and ¥ indicating simple main effects. Results support a domain general difficulty with visual conjunctive processing in ASD. Error bars represent standard error. Panel **(G)** depicts the relationship between autistic traits and conjunctive processing. Higher levels of autistic traits were associated with lower levels of conjunctive processing. For all bar graphs, TD individuals are shown in black/gray and ASD individuals in red/pink. Low-ambiguity conditions (LA) are presented in red/black, and high-ambiguity conditions (HA) are presented in pink/gray. Outliers are indicated with +, but in each case inclusion of outliers did not change the significance of results.

We predicted that TD individuals would process high-ambiguity objects more conjunctively than low-ambiguity objects (Barense et al., 2012; Newsome et al., 2012). Faces are an inherently conjunctive stimulus class even at low levels of ambiguity, and thus were predicted to be processed in an equally conjunctive manner regardless of ambiguity level (Tanaka and Farah, 1993; Rossion, 2008; Van Belle et al., 2010; Behrmann et al., 2015). Figure 2A shows a graphical representation of these hypotheses in the TD group. If atypical visual conjunctive processing in ASD is specific to faces, we would predict no change in the pattern or overall level of conjunctive processing with objects but would see a reduction in conjunctive processing of faces (Figure 2B). A domain-general change in conjunctive processing could arise in two distinct patterns. First, an overall reduction in conjunctive processing relative to TD regardless of stimulus type would be predicted by the weak central coherence hypothesis (Happe, 1999; Happe and Frith, 2006). This would manifest as a reduction in the absolute amount of conjunctive processing, while preserving the pattern of differences in conjunctive processing across stimulus type (Figure 2C). Alternatively, the ASD group may show a lack of perceptual specialization whereby all stimuli are processed with a similar level of conjunctive processing, whether they are faces or non-face objects, and regardless of ambiguity level (Hadad et al., 2017). This would manifest as a change in the pattern of differences in conjunctive processing across stimulus types, but not necessarily an absolute reduction (Figure 2D).

## Methods

### Participants

Sixty-three individuals participated, 53 TD (mean age = 14.1 ± 5.1 years, range = 6–24 years) and 10 autistic individuals (mean age = 17.9 ± 6.6 years, range = 9–32 years). Groups were matched for performance IQ using the Wechsler Abbreviated Scale of Intelligence two matrix reasoning subtest *t*-scores (ASD mean = 47.7 ± 14.6, TD mean = 52.4 ± 9.0). Participants had self-reported normal or corrected-to-normal vision. Autistic participants provided written documentation of diagnosis, which were confirmed by administration of the Autism Diagnostic Observation Schedule. All participants provided informed, written consent, and all protocols were approved by the University of Toronto Research Ethics Board, and were aligned with the Declaration of Helsinki.

### Stimuli

Faces and objects were presented in high- and low-ambiguity levels extracted from the behavioral pilot (see Supplementary Material). High-ambiguity face pairs were morphed such that they differed by 10%, whereas low-ambiguity face pairs differed by 60%. Object stimuli have been thoroughly described previously (Figure 1 and Barense et al., 2012).

### Eye-Tracking Task

Each condition (high- and low-ambiguity objects and faces) was presented in a blocked design, with 72 trials per condition. Stimuli were presented in pairs, and participants identified via button press if the faces/objects were the same or different, with 36 trials of each. Six practice trials with feedback were presented prior to each block. Block orders were counterbalanced across participants. Trials were self-paced with a maximum of 15 s per stimulus pair or a button response terminating a trial. Eye movements were measured using an Eyelink 1000 monocular system (SR Research) and sampled at 1000 Hz and a spatial resolution of 0.1°.

### Analysis

Conjunctive versus feature-based strategies were assessed by comparing the number of saccades an individual made within an object/face versus the number of saccades between objects/faces in non-match trials (within:between ratios). The ratio of within- to between-item saccades was calculated on an individual trial basis, and averaged across all trials in each condition:

$$\begin{array}{lll} {\left(\frac{\text{Within}}{\text{Total}}\right)}_{\text{Avg}} & = & \frac{\frac{W_{1}}{T_{1}}+\frac{W_{2}}{T_{2}}+\cdots \frac{W_{36}}{T_{36}}}{36}:\frac{\frac{B_{1}}{T_{1}}+\frac{B}{T_{2}}+\cdots \frac{B_{36}}{T_{36}}}{36}\\ & = & {\left(\frac{\text{Between}}{\text{Total}}\right)}_{\text{Avg}} \end{array}$$

The higher the ratio of within:between saccades, the greater the level of conjunctive processing (Figure 1).

A three-way, mixed-model ANOVAs (diagnostic group × stimulus type × ambiguity) was conducted examining saccade patterns. Given the difference in sample sizes, ANOVAs are only robust when the assumption of homogeneity of variance is met. To ensure this Levene’s tests were conducted, and this assumption was met for all conditions without exception, ensuring the validity of the ANOVA (low-ambiguity objects, *F*_(1,61)_ = 0.001, *p* = 0.97; high-ambiguity objects, *F*_(1,61)_ = 0.58, *p* = 0.45; low-ambiguity faces, *F*_(1,61)_ = 0.002, *p* = 0.96; high-ambiguity faces, *F*_(1,61)_ = 0.42, *p* = 0.52). Follow-up 2-way ANOVAs and *t*-tests were conducted where significant interactions were found.

### Correlations With Autistic Traits

Fifty-nine participants completed the Autism-Spectrum Quotient (Baron-Cohen et al., 2001) to explore the relationship between conjunctive processing and autistic traits, with the *a priori* hypothesis that individuals showing greater levels of autistic traits would exhibit less conjunctive processing overall.

## Results

### Accuracy

Results confirmed that face and object stimuli were well matched, with faces and objects within 5% accuracy of each other in both the high- and low-ambiguity conditions (high-ambiguity faces = 0.63 ± 0.17 and objects = 0.67 ± 0.17; low-ambiguity faces = 0.87 ± 0.11 and objects = 0.91 ± 0.07). To ensure these differences did not vary across ambiguity levels, a 2-way ANOVA was conducted, and no significant stimulus-by-ambiguity interaction was observed (*F*_(1,1)_ = 0.02, *p* = 0.89).

### Eye-Tracking

The omnibus ANOVA of within:between saccade ratios revealed a significant main effect of ambiguity: High-ambiguity stimuli induced greater conjunctive processing than low-ambiguity stimuli (*F*_(1,61)_ = 148.59, *p* < 0.001, η_p_^2^ = 0.71) (Figures 2E,F). No main effect was observed for stimulus type (*F*_(1,61)_ = 3.45, *p* = 0.07, η_p_^2^ = 0.05), or diagnostic group (*F*_(1,61)_ = 0.21, *p* = 0.65, η_p_^2^ < 0.01). A significant stimulus-type-by-ambiguity interaction was observed, with a greater difference between high- and low-ambiguity conditions occurring in object stimuli (*F*_(1,61)_ = 8.48, *p* = 0.005, η_p_^2^ = 0.12). Likewise, a group-by-ambiguity interaction was observed, with the TD group exhibiting a greater impact of ambiguity level than the ASD group (*F*_(1,61)_ = 3.88, *p* = 0.05, η_p_^2^ = 0.06). No significant group-by-stimulus type interaction was observed (*F*_(1,61)_ < 0.01, *p* = 0.99, η_p_^2^ < 0.01). Importantly, a 3-way group-by-stimulus type-by-ambiguity interaction was observed (*F*_(1,61)_ = 4.33, *p* = 0.04, η_p_^2^ = 0.07). To understand what was driving this 3-way interaction, two pairs of 2-way ANOVAs were conducted (see Supplementary Material) confirming that this 3-way interaction was driven by a lack of perceptual specialization in the ASD group. This lack of difference across all conditions in the ASD group was confirmed using Bayesian RM ANOVAs, not only showing a lack of significant perceptual specialization, but showing evidence *for* no perceptual specialization.

### Correlations With Autistic Traits

A Pearson correlation was conducted to explore the relationship between conjunctive processing and autistic traits, demonstrating that individuals showing greater levels of autistic traits exhibited less conjunctive processing overall (Figure 2G, *r*_(58)_ = 0.30, *p* = 0.02, CI = [0.05–0.51]). Note that with the removal of the most extreme participant (AQ = 41, correlational results do not differ (*r*_(57)_ = 0.28, *p* = 0.03, CI = [0.02–0.50]).

## Discussion

This study tested whether atypical face processing in autism related to differences with visual conjunctive processing, and whether these were face-specific or domain-general effects. TD participants showed increases in visual conjunctive processing with ambiguous relative to unambiguous objects, whereas conjunctive processing of faces was not modulated by ambiguity. Autistic individuals did not follow this pattern: They did not modulate their degree of conjunctive processing based on ambiguity level or stimulus category. Further, higher levels of autistic traits were associated with reduced levels of conjunctive processing.

These preliminary results support accounts of atypical visual conjunctive processing in ASD, demonstrating that differences with conjunctive processing are present in, but not limited to, processing of faces. Indeed, the clearest difference between TD and ASD participants’ gaze patterns was observed in the autistic individuals’ lack of an ambiguity effect when processing object stimuli. These results align with perceptual accounts of autism, such as Enhanced Perceptual Functioning (Mottron and Burack, 2001; Mottron et al., 2006) and Weak Central Coherence (Happe, 1999; Happe and Frith, 2006). These accounts postulate that autistic individuals default to a locally oriented perceptual and cognitive style, possibly at the expense of globally oriented processing. Thus, impairments in face identification may be due to a domain-general difference in perceptual strategy rather than a specific impairment in face processing. In the current study, this would present as a feature-based relative to a conjunctive-processing strategy. With that said, individuals with ASD did *not* show an overall decrease in conjunctive processing as predicted by Weak Central Coherence, but showed similar levels of conjunctive processing across stimulus type or ambiguity levels. This finding falls in line with a recent hypothesis that autistic individuals showed a lack of *perceptual specialization* (Hadad et al., 2017). Perceptual specialization refers to the tuning of perceptual strategies or processes across development to facilitate a particular task or the processing of particular stimuli (Johnson, 1999, 2000). Our data suggest a lack of perceptual specialization in the form of atypical visual conjunctive processing in ASD, whereby autistic individuals do not utilize a more conjunctive-processing strategy for high- relative to low-ambiguity objects or faces. This lack of perceptual specialization of visual conjunctive processing may contribute to face-perception difficulties in autism, and possibly compounding additional difficulties processing social information. Given that the use of a conjunctive-processing strategy for face perception results in a faster and more accurate perception of faces in typical individuals, a failure to perceptually specialize toward conjunctive processing of faces may present as a specific deficit in face perception in autism.

These preliminary results provide an avenue for future exploration of conjunctive visual processing in ASD. Also, the successful implementation of this paradigm provides a possible means to test visual conjunctive processing in autistic individuals with lower-functioning levels. While behavioral responses were collected in the current study, the eye-tracking component and analyses could be implemented passively in children who are unable to perform tasks with cognitive demands or follow verbal instructions beyond maintaining attention to a screen.

Given the low number of participants in this initial preliminary cohort, it will be important to replicate with a large cohort. Additional research into visual conjunctive processing in ASD should include a larger number of children, adolescents, and adults, as there have been multiple studies of perception of social stimuli that show that the differences observed between TD and ASD participants changes throughout development (Taylor et al., 2010; Stevenson et al., 2014b).

## Author Contributions

RS, NH, L-KY, SF, and MB were involved with study design. RS drafted the manuscript. All authors were involved with data collection, analysis, and interpretation of results and also edited the manuscript.

## Conflict of Interest Statement

The authors declare that the research was conducted in the absence of any commercial or financial relationships that could be construed as a potential conflict of interest.
