# Supplementary material for: Conjunctive Visual Processing Appears Abnormal in Autism
Source: Front Psychol. 2019 Jan 18;9:2668. doi: 10.3389/fpsyg.2018.02668 (PMC6346680; doi:10.3389/fpsyg.2018.02668)
Supplement: Supplementary file 1 [file Data_Sheet_1.pdf]

Supplemental Materials

Behavioural pilot. The behavioural pilot to match stimulus types for difficulty included 15 TD undergraduate students (mean age=19.0±1.7 years, range=18-23 years). We presented two categories of stimuli (abstract objects and faces) each at two levels of visual ambiguity (low and high). For all stimulus presentations, stimuli subtended a visual angle ranging from 5.45-9.15°.

Object stimuli were taken from previous studies testing visual ambiguity (Barense et al., 2005; Barense et al., 2012), and consisted of three explicitly-defined component features; outer shape, inner shape, and fill (Figure 1). For the high-ambiguity stimulus pairs, two of the three components were identical. For low-ambiguity stimulus pairs, all three components were different. All stimulus pairs were presented with randomly-varied rotations between 15 and 165°. Face stimuli were constructed using FaceGen, and included whole faces and necks, with no hair. As has been successfully implemented in past studies (Lee et al., 2005), we morphed faces to create varying levels of feature ambiguity from 0-100% in 10% increments. All face pairs were presented with one face rotated 20° to the left or right, a rotation that has previously been identified to induce face-perception difficulties in autism (Morin et al., 2015).

Participants completed a same-different matching task on both object and face stimuli. Psychophysical thresholds were calculated by fitting a sigmoid curve to each individual participant's accuracy rates. Two thresholds were then extracted from these individual face-based psychometric functions: one that most closely matched their accuracy with high-ambiguity objects, and one that most closely matched low-ambiguity objects, such that difficulty was matched across stimulus types for each level of ambiguity in the primary experiment. Results showed that the accuracy achieved with the 60% face morphs matched accuracy with the low-ambiguity objects and 90% face morphs matched accuracy with the high-ambiguity objects.

Follow-up analyses with eye-tracking data. Splitting data by diagnostic group, follow-up 2-way ANOVAs (stimulus type x ambiguity) were then conducted on the ASD and TD groups individually. In TD participants, a significant stimulus x ambiguity interaction was observed ($F_{(1,61)}$=41.91, p<0.001, η_p^2=0.45), which was driven by a significant effect of ambiguity with objects (t=8.20, p<0.001, d=0.89), with no such significant effect with faces (t=1.45, p=0.15, d=0.14) (Figure 3A, gray/black, interaction indicated by #). A main effect of ambiguity was observed, again with more ambiguous stimuli being processed in a more conjunctive manner ($F_{(1,52)}$=35.89, p<0.001, η_p^2=0.41). This main effect should be interpreted cautiously, however, given the interaction between stimulus type and ambiguity reported above. No main effect of

stimulus type was observed ($F_{(1,52)=}$0.16, p=0.15, η_p^2=0.04). The follow-up 2-way ANOVA (stimulus x ambiguity) in the ASD group revealed no main effect of either ambiguity ($F_{(1, 9)=}$0.49, p=0.50, η_p^2=0.05) or stimulus type ($F_{(1, 9)<}$0.01, p=0.99, η_p^2<0.01), and no interaction between the two ($F_{(1,9)=}$0.15, p=0.71, η_p^2=0.02) (Figure 2E, red/pink). Given the lack of interaction, no follow-up t-tests were conducted.

Splitting data by stimulus type, follow-up 2-way ANOVAs (diagnostic group x ambiguity) were then conducted on the object and face conditions individually to explore what was driving the 3-way interaction. With object stimuli (Figure 2E), a main effect of ambiguity was observed, again with more ambiguous stimuli being processed in a more conjunctive manner ($F_{(1,61)=}$14.48, p<0.001, η_p^2=0.19). No main effect of diagnostic group was observed ($F_{(1,61)=}$0.05, p=0.82, η_p^2<0.01). A significant diagnostic group x ambiguity interaction was observed ($F_{(1,61)=}$6.79, p=0.01, η_p^2=0.10), though no significant differences of diagnosis was seen with high-ambiguity ($t_{(61)}$=1.09, p=0.28, d=0.41) or low-ambiguity objects ($t_{(61)}$=1.15, p=0.25, d=0.38). Thus, the group x ambiguity interaction is driven by the fact that TD individuals modulated their conjunctive processing based on the ambiguity levels of the object, whereas ASD individuals did not. The follow-up 2-way ANOVA (diagnostic group x ambiguity) in the face conditions (Figure 3A) revealed no main effect of either ambiguity ($F_{(1,61)=}$0.63, p=0.43, η_p^2=0.01) or diagnostic group ($F_{(1,9)<}$0.36, p=0.55, η_p^2<0.01), and no interaction between the two ($F_{(1,9)=}$0.19, p=0.67, η_p^2<0.01). Given the lack of interaction, no follow-up t-tests were conducted.

Given the null effects found in the ASD group, a follow-up, Bayesian RM ANOVA was conducted across ambiguity level and stimulus type. Both main effects of ambiguity level and stimulus type were over three times more likely to fit the null model that showing significant main effects (BFs = 0.3), and the model including an ambiguity-by-stimulus interaction was 25 times more likely to support the null model (BF = 0.04).

Age effects. Given the age range of the samples, effects of age were analyzed given previously reported differences observed between TD and ASD participants throughout development (Taylor et al., 2010; Stevenson et al., 2014). To explore such a possible age-effect, a "double difference" comparing the impact of ambiguity on objects and faces was calculated for each individual [($Object_{HA}$- $Object_{LA}$) – ($Face_{HA}$- $Face_{HA}$)]. Positive values indicate a greater impact of ambiguity on object perception than on faces, and vice versa for negative values. TD individuals averaged a double difference of 0.33±0.37, significantly greater than the ASD average of 0.05±0.45 (t_{61}=2.08, p=0.04). This indicates that TD participants were more likely than autistic individuals to show a greater ambiguity effect with objects than with faces.

Individual's double differences were then correlated with age. Neither TD ($r_{(62)}$=0.03, p=0.83) nor ASD ($r_{(62)}$=-0.08, p=0.83) individuals' patterns of conjunctive processing were related to age.

Barense, M.D., Bussey, T.J., Lee, A.C., Rogers, T.T., Davies, R.R., Saksida, L.M., et al. (2005). Functional specialization in the human medial temporal lobe. *Journal of Neuroscience* 25(44)**,** 10239-10246.

Barense, M.D., Groen, I.I., Lee, A.C., Yeung, L.-K., Brady, S.M., Gregori, M., et al. (2012). Intact memory for irrelevant information impairs perception in amnesia. *Neuron* 75(1)**,** 157-167.

Lee, A.C., Bussey, T.J., Murray, E.A., Saksida, L.M., Epstein, R.A., Kapur, N., et al. (2005). Perceptual deficits in amnesia: challenging the medial temporal lobe 'mnemonic'view. *Neuropsychologia* 43(1)**,** 1-11.

Morin, K., Guy, J., Habak, C., Wilson, H.R., Pagani, L., Mottron, L., et al. (2015). Atypical face perception in autism: A point of view? *Autism Research* 8(5)**,** 497-506.

Stevenson, R.A., Siemann, J.K., Woynaroski, T.G., Schneider, B.C., Eberly, H.E., Camarata, S.M., et al. (2014). Brief report: arrested development of audiovisual speech perception in autism spectrum disorders. *J Autism Dev Disord* 44(6)**,** 1470-1477. doi: 10.1007/s10803-013-1992-7.

Taylor, N., Isaac, C., and Milne, E. (2010). A comparison of the development of audiovisual integration in children with autism spectrum disorders and typically developing children. *J Autism Dev Disord* 40(11)**,** 1403-1411. doi: 10.1007/s10803-010-1000-4.
